# Evaluation of Tanshinone IIA Developmental Toxicity in Zebrafish Embryos

**DOI:** 10.3390/molecules22040660

**Published:** 2017-04-21

**Authors:** Tao Wang, Chengxi Wang, Qiong Wu, Kangdi Zheng, Jiaojiao Chen, Yutao Lan, Yao Qin, Wenjie Mei, Baoguo Wang

**Affiliations:** 1 School of Public Health, Guangdong Pharmaceutical University, Guangzhou 510310, Guangdong, China; wangtao1234@126.com; 2 School of Pharmacy, Guangdong Pharmaceutical University, Guangzhou 510006, Guangdong, China; gdpuwcx@163.com (C.W.); 13533260720@163.com (Q.W.); kangdizheng@163.com (K.Z.); 3 School of Nursing, Guangdong Pharmaceutical University, Guangzhou 510310, Guangdong, China; ch381286034@126.com; 4 Chn-Alternative Biotech Co. Ltd., Guangzhou 510432, Guangdong, China; chnalt@163.com

**Keywords:** zebrafish, tanshinone IIA, developmental toxicity

## Abstract

Tanshinone IIA (Tan-IIA) is derived from the dried roots of *Salvia miltiorrhiza* Bunge, a traditional Chinese medicine. Although *Salvia miltiorrhiza* has been applied for many years, the toxicity of the mono-constituent of *Salvia miltiorrhiza*, tanshinone IIA, is still understudied. This study evaluated the cardiotoxicity and developmental malformations of Tan-IIA by using zebrafish normal embryos and dechorionated embryos. After treatment with Tan-IIA in different concentrations for four-day periods, obvious pericardial edema, spinal curvature, and even missing tails were observed in zebrafish embryos. The LC_50_ values in the dechorionated embryo group at 72 h post-fertilization (hpf) and 96 hpf were 18.5 μM and 12.8 μM, respectively, and the teratogenicity was manifested at a concentration of about 1 µM. The main endpoints of teratogenicity were scoliosis, malformation of tail, and pericardium edema. Our findings displayed the potential cardiotoxicity and severe impact on the abnormal development of Tan-IIA in zebrafish embryo at high concentrations, which may help avoid the risk of its clinical application.

## 1. Introduction

Tanshinone IIA (Tan-IIA), a fat-soluble fuchsia needle crystal, is the main active ingredient of diterpene quinone in traditional Chinese medicine *Salvia miltiorrhiza*. Because of its unique quinoid structure, Tan-IIA may be involved in multiple biochemical reactions of the organism and have a variety of biological activities. Therefore, Tan-IIA is widely used in the treatment of cardiovascular disease [1,2,3]. A large amount of researches show that Tan-IIA exhibits various pharmacological activities, including anti-inflammatory [4], anti-oxidative [5], anti-fibrosis [6], modulation of collagen metabolism [7], anti-tumor [8] and so on.

Zebrafish (*Danio rerio*) is a small tropical fish with the model advantages of large spawning, rapid breeding, and being easy to raise, etc. [9]. It has been recommended as an animal model for some standard toxicology tests. Evaluation of drug toxicity using zebrafish has the advantages of easy observation, low cost, simple operation, good repeatability, high sensitivity, and short experiment periods, and is an internationally recognized standard method of evaluation of drug toxicity [10]. Zebrafish embryo developmental toxicity is based on teratogenesis and mortality of the zebrafish embryos [11,12]. The current acute toxicity experiments are mainly concentrated in mice as a representative of the animal model. However, due to its high breeding conditions, high cost, complex operation, and ethical limits, it is difficult to achieve comprehensive urgent toxicity tests with this model [13,14]. Therefore, using the zebrafish model to evaluate drug toxicity is of great significance, and the zebrafish model has been widely used in the assessment of acute toxicity and developmental toxicity [15,16,17,18,19,20].

In a previous study, our group explored the acute toxicity of dimethyl sulfoxide (DMSO) by using the zebrafish embryo model [21], which could thus be utilized for choosing the safe concentration range of DMSO as a solvent [22]. This current study firstly investigated the developmental toxicity effect of Tan-IIA on the zebrafish embryo model. The proposed zebrafish acute toxicity assay is very valid and reliable for rapid evaluation of Tan-IIA toxicity, and saves time and cost during drug research and development.

## 2. Results

### 2.1. The Crystal Structure of Tan-IIA

**Figure 1:**
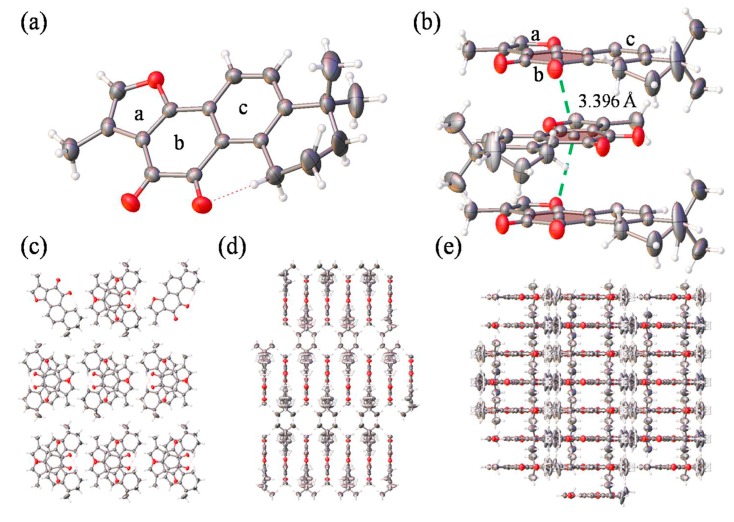
(**a**) The molecular structure of Tan-IIA; (**b**) Stacking interactions in a step showing strong overlap for Tan-IIA. The step showed two benzene rings with a diketone group stacking interactions occurring with distances to the ring centroid of 3.396 Å. The pores viewed along the a axis (**c**), b axis (**d**) and c axis (**e**).

Single crystal X-ray diffraction analysis revealed that Tan-IIA crystallized in the Pmna space group, and the data is summarized Table S1. The fundamental asymmetric group contained only one Tan-IIA molecule. As shown in Figure 1a, two dibenzopyrrole units displayed a large aromatic planar structure with one furan ring (ring a) and two benzene ring (ring b and c), which the hexamethylene group showed a classic chair conformation. Stacking interactions in a step showed strong overlap for Tan-IIA. As shown in Figure 1b, it is showed great inter-molecular interaction between two benzene rings with a diketone group (ring b) of two molecules occurring with distances to the ring centroid of 3.396 Å by π-π stacking interactions. This created an infinite 1D ribbon composed of Tan-IIA secondary building units (SBUs) running along the b axis, as seen in Figure 1d. The layers consisted of a benzene ring with a diketone group positioned adjacent to each other, separated by 3.396 Å via their π-π stacking interactions, as seen in Figure 1d,e.

### 2.2. The Lethal Effects of Tan-IIA on Embryos

**Figure 2:**
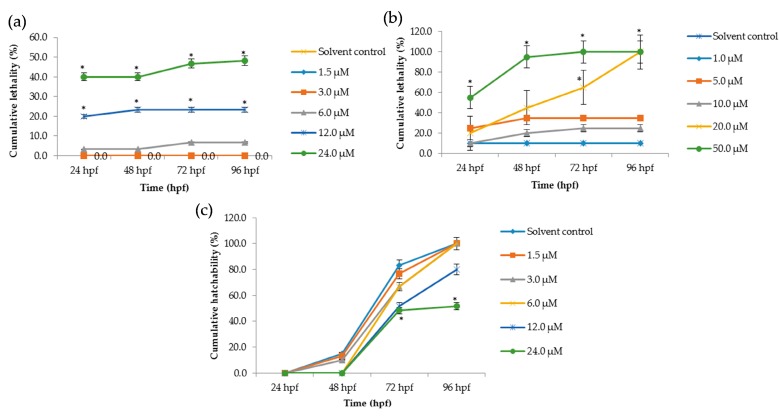
Cumulative lethality and hatchability curves of embryos exposed to different concentrations of Tan-IIA. (**a**) Cumulative lethality curves of chorionic embryos; (**b**) Cumulative lethality curves of dechorionated embryos; (**c**) Cumulative hatchability curves of chorionic embryos. (*n* = 20 zebrafish per treatment; * *p* < 0.05, compared to control).

The lethal effects of Tan-IIA on zebrafish embryos were recorded at 24 hpf, 48 hpf, 72 hpf and 96 hpf, as shown in Figure 2, and at 12 hpf as shown in Tables S2 and S3. In the chorionic embryo group, compared with the control group, little obvious lethal effect at concentrations of 1 μM and 3 μM was observed, but with the increasing concentration of Tan-IIA treatment, severe abnormalities of heart and pericardium occurred, which were observed in a dose-dependent manner. The mortality at 24 μM for 96 hpf was just 51.6% (Figure 2a). Meanwhile in the dechorionated embryo group, lethal effect appeared at a concentration of 5 μM, even reaching 100% mortality (50 μM at 72 hpf and 20 μM at 96 hpf, as shown in Figure 2b). The LC_50_ values in the dechorionated embryo group at 72 hpf and 96 hpf were 18.5 μM and 12.8 μM, respectively. The hatchability of the chorionic embryo group is shown in Figure 2c, which indicated that high concentrations of Tan-IIA (12 μM, 24 μM) affected the hatchability of the embryos. In the dechorionated embryo group, the hatchability could not be calculated due to the chorion being removed. The main lethal endpoints observed in the dechorionated embryo group were coagulated embryos at 48 hpf and lack of heartbeat at 96 hpf; lack of somite formation and non-detachment of the tail were also observed in some cases. The abovementioned results indicated that Tan-IIA exhibited some toxicity to zebrafish embryos in a dose-dependent manner.

### 2.3. The Teratogenic Effects of Tan-IIA on Embryos

**Figure 3:**
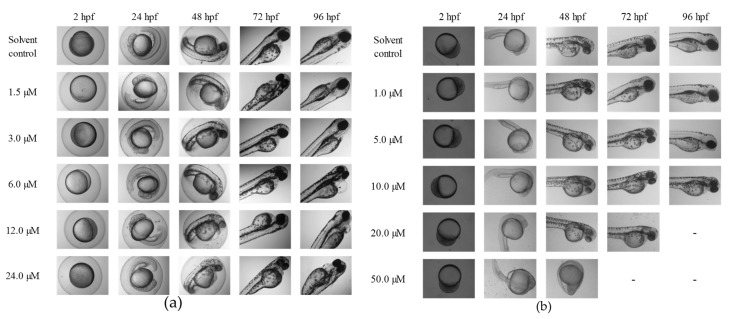
Morphology of zebrafish embryos exposed to Tan-IIA. (**a**) Morphology of chorionic embryos; (**b**) Morphology of dechorionated embryos—denotes that the treated embryos were all dead.

The morphological changes of zebrafish embryos induced by Tan-IIA were further evaluated. Zebrafish have rapidly developed into a promising model for whole-organism toxicology screening. The influence of Tan-IIA on the development of Zebrafish embryos was determined, as shown in Figure 3 and Figure S1 (12 hpf), and the detailed data are listed in Table S4.

As shown in Figure 3a, in the chorionic embryo group, the zebrafish embryos without Tan-IIA treatment had developed normally, but following treatment with an increasing dosage of Tan-IIA, the hatched fish developed obvious pericardial edema at 6 μM for 96 hpf and spinal curvature at 24 μM for 96 hpf. Moreover, after treatment with Tan-IIA ≥ 6 μM, the time it took for the embryos to mature into fish was longer than that for the control group, which suggested that Tan-IIA exhibited growth inhibition of zebrafish embryos. Furthermore, in the dechorionated embryo group, the zebrafish embryos without chorions could uptake larger amounts of drugs. It was found that the embryos all died at 20 μM for 96 hpf and 50 μM for 72 hpf, and the growth of embryos were inhibited seriously at 50 μM. In a word, above results suggested that Tan-IIA, in a concentration-dependent and low time-dependent manner, exhibited certain toxicity and growth inhibition in zebrafish embryos in vivo at high concentrations.

**Figure 4:**
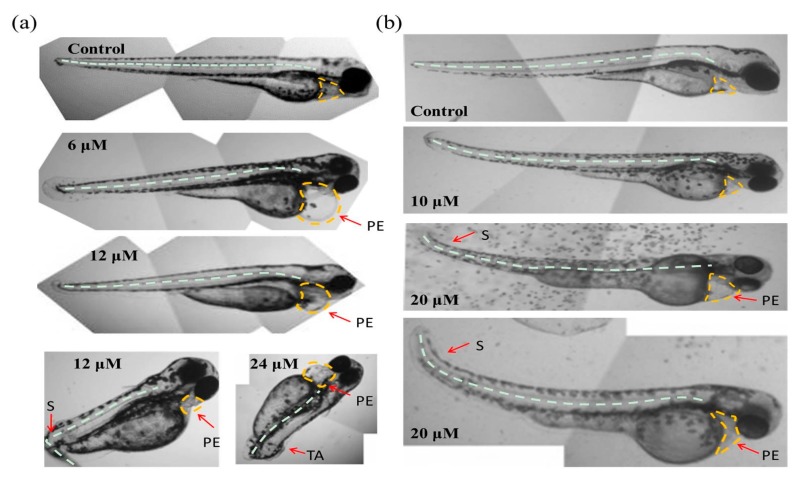
Abnormal embryos exposed to Tan-IIA. (**a**) Abnormal embryos in the chorionic embryo group; (**b**) Abnormal embryos in the dechorionated embryo group. S: scoliosis; PE: pericardial edema; TA: tail autolysis.

Moreover, with the protection of the chorion, the zebrafish hatched from eggs exposed to different concentrations (0, 1.5 and 3 μM) of Tan-IIA for 96 hpf displayed great health conditions, but the zebrafish treated with 6 μM showed abnormal development with pericardial edema and scoliosis, as well as some with the tail missing at 24 μM. However, without the protection of the chorion, the zebrafish were subjected to mass mortality and mild teratogenic effects at 5 μM for 96 hpf. All of the major malformations of scoliosis, tail autolysis, and pericardial edema were observed at the highest concentration of 50 μM (Figure 4b). Especially in groups treated with 20 μM and 50 μM of Tan-IIA, the zebrafish pericardium was enlarged, edematous and congested, which appeared to be about two-times larger than that of the control group. The longitudinal sections of pericardial edema and scoliosis with hematoxylin-eosin (HE) staining were shown in Figure S2. These results indicated that Tan-IIA exhibited potential cardiotoxicity and growth inhibition in zebrafish embryos.

## 3. Discussion

Although *Salvia miltiorrhiza* has been applied for many years, the toxicity of the mono-constituent of *Salvia miltiorrhiza*, tanshinone IIA, has not been fully studied. Cao Xiaomei et al. [23] used cavy and rabbit to evaluate the hemolytic effect, hormesis effect and anaphylaxis of sodium tanshinone IIA sulfonate injection. Our research explored the developmental toxicity and acute toxicity of Tan-IIA by using chorionic and dechorionated zebrafish embryos. The lethality and teratogenicity were observed and summarized at different concentrations of Tan-IIA.

The chorion of a zebrafish embryo is the acellular envelope surrounding embryo. The role of the chorion is to protect the embryo from damage and to prevent polyspermy [24,25]. As shown in Figure 2a,b, the lethality of the chorionic embryo group was lower than dechorionated embryo group at similar concentrations of Tan-IIA. In the dechorionated embryo group, the LC_50_ values at 72 hpf and 96 hpf were 18.5 μM and 12.8 μM, respectively, while in the chorionic embryo group, the lethality of the highest concentration (24 μM) was below 50%. These results indicated that Tan-IIA may be partly blocked by the chorion. The incubation of the zebrafish embryo is the result of hatching enzymes and mechanical force [26]. In the process of hatching, the chorion is decomposed by hatching enzymes and then the embryo, with warp and sway movements, would break through the chorion [27,28]. As shown in Figure 2b, the hatchability of the embryos declined at a concentration of 12 μM and 24 μM, which revealed that Tan-IIA in high concentrations might affect the functions of hatching enzymes and/or the motor system of the embryo.

The development of zebrafish is very similar to mammals in most aspects of embryo development, including early embryonic processes and the development of cardiovascular, somite and skeletal structures, etc. [11,12]. When the embryos were exposed to chemicals upon fertilization, teratogenic effects could be observed in the following several days. As shown in Figure 4 and Table S4, there were no teratogenic effects when the concentration of Tan-IIA was below 5 μM in both the chorionic and dechorionated embryo groups. The major malformations observed were scoliosis, tail autolysis, and pericardial edema. Due to the resistance function of the chorion, the appearance of teratogenic effect was delayed compared to the dechorionated embryo group.

## 4. Materials and Methods

### 4.1. Materials and Reagents

Tanshinone IIA (Tan-IIA) was purchased from Damao Chemical Reagent Factory (Tianjin, China), protease E from Sigma-Aldrich (Guangzhou, China), dimethyl sulfoxide (DMSO) from Guangzhou Chemical Reagent Factory (Guangzhou, China), the 6-well plate from Guangzhou JET Bio-Filtration Co., Ltd. (Guangzhou, China), and the inverted optical microscope from Chongqing Optec Instrument Co., Ltd. (Chongqing, China).

### 4.2. Brood Zebrafish Maintenance and Egg Production

Zebrafish were maintained according to *The Zebrafish Book* [29]. Wild type Tuebingen strain zebrafish (*Danio rerio*) were obtained from China Zebrafish Resource Center, CZRC (Wuhan, China), and three-month-old zebrafish were used for egg production. Sexually mature zebrafish were maintained in a recirculating aquaculture system (Shanghai Haisheng Biotech Co., Ltd, Shanghai, China) at 28.0 ± 1.0 °C. The water was purified by water purifier (Shenzhen Luoke Water Purification Equipment Co., Ltd, Shenzhen, China), and NaHCO_3_ and NaCl were used to adjust the pH and conductivity at pH 6.8~7.4 and 500~550 µS, respectively. The housing system was equipped with a temperature control unit, UV light, and activated carbon filter system. The day to night photoperiod was 14 h: 10 h. Mature zebrafish were fed with brine shrimp twice and powder feed once daily.

Males and females at a ratio of 2:1 or 1:1 were placed in spawning tanks a few hours before the onset of darkness on the day prior to the test. For the collection of eggs, spawn traps were placed into the spawning tanks, and the spawn traps were covered with inert wire mesh of appropriate mesh size. About 30 min after the onset of light, spawn traps were removed and eggs were collected. To avoid genetic bias, eggs were collected from a minimum of three breeding groups, mixed and randomly selected.

### 4.3. The Developmental Toxicity Assay

The developmental toxicity assay was carried out according to the fish embryo acute toxicity (FET) test, developed from Organization for Economic Cooperation and Development (OECD) guidance [30] and the book entitled *Zebrafish: Methods for Assessing Drug Safety and Toxicity* with some modifications [31,32].

#### 4.3.1. Removal of Chorion

The method of removing the chorion was taken from the published protocol [33]. Eggs were placed in a 90 mm petri dish and the water was sopped up. 5 mL of pronase (0.208 g/L, warmed to 28.5 °C) was poured into the petri dish and incubated for 6 min. 20 mL fish water was added to the petri dish, and the eggs were allowed to sink to the bottom of the petri dish before the water was slowly poured out. The eggs were gently rinsed three times with fish water and most of the chorions had thus been removed.

#### 4.3.2. Embryo Exposure

In the preliminary experiment, the concentrations of Tan-IIA of 1.5 µM, 3.0 µM, 6.0 µM, 12.0 µM, 24.0 µM were selected. Tan-IIA was dissolved in DMSO with a final solvent concentration of 0.2% in the test solution. 5 mL of these solutions and 20 chorionic embryos of 2 h post-fertilization (hpf) were transferred to 6-well plates. Each test concentration and solvent control were on the same plate, amounting to three parallel experimental plates and one negative control plate of fish water. The embryos were incubated at 28.0 ± 1.0 °C with the photoperiod of 14 h:10 h.

According to the results of the pre-test, the concentrations of Tan-IIA were changed to 1.0 µM, 5.0 µM, 10.0 µM, 20.0 µM, and 50.0 µM, with the final DMSO concentration of 0.4% in the test solution. In addition, normal dechorionated embryos of 2 h post-fertilization (hpf) were used for exposure. The other methods were the same as the preliminary experiment described above.

#### 4.3.3. Observation of Mortal and Teratogenic Effects

Observations were recorded using an inverted optical microscope every 24 h until the end of the test, including coagulation of embryos, lack of somite formation, non-detachment of the tail, and lack of heartbeat. Any positive outcomes in these observations indicated that the zebrafish embryo was dead. The teratogenic embryos were photographed to analyze the deformed parts. LC_50_ were calculated at 72 hpf and 96 hpf.

### 4.4. Statistical Analysis

The significance of mortality and hatching rates were determined by chi-square (χ^2^) test and Fisher’s exact test, because both death and hatching are categorical variables. Significant differences were considered at *p* < 0.05. Statistical analysis was conducted using SPSS 17.0 (SPSS Statistics 17.0.1, International Business Machines Corp., Armonk, NY, USA).

## 5. Conclusions

Tanshinone IIA, the main active ingredient of S*alvia miltiorrhiza*, is an important drug to treat diseases of cardiovascular system such as hypertension and atherosclerosis. The in vitro potential toxicity of Tan-IIA was evaluated by the zebrafish embryo model. It was found that Tan-IIA exhibited severe growth inhibition, development malformation and cardiotoxicity at high concentrations. Sodium tanshinone IIA sulfonate injection has clinical applications at a dose of 40–80 mg per day, which is a low concentration compared to those investigated in this study applied to zebrafish. Therefore, sodium tanshinone IIA sulfonate injection may cause minimal side effects at a clinical dose, which is consistent with the results of clinical research [34]. This study firstly reported the potential toxicity of Tan-IIA at high concentrations on zebrafish, pointing out the potential risk of its clinical application at an increased dose.

## Supplementary Materials

Supplementary materials are available online, including Figures S1 and S2.
