# Supplementary material for: Evaluation of Tanshinone IIA Developmental Toxicity in Zebrafish Embryos
Source: Molecules. 2017 Apr 21;22(4):660. doi: 10.3390/molecules22040660 (PMC6154573; doi:10.3390/molecules22040660)
Supplement: Supplementary file 1 [file molecules-22-00660-s001.pdf]

# Supplementary Materials: Evaluation of Tanshinone IIA Developmental Toxicity in Zebrafish Embryos

**Table S1.** Crystal data and structure refinement for (Tanshinone IIA).

| | |
|---|---|
| Empirical formula | $C_{19}H_{18}NO_3$ |
| Formula weight | 294.33 |
| Temperature/K | 100(2) |
| Crystal system | N/A |
| Space group | Pmna |
| a/Å | 6.646(3) |
| b/Å | 9.193(4) |
| c/Å | 24.468(10) |
| $\alpha$/° | 90.00 |
| $\beta$/° | 90.00 |
| $\gamma$/° | 90.00 |
| Volume/Å$^3$ | 1494.9(11) |
| Z | 4 |
| $\rho_{calc}$g/cm$^3$ | 1.308 |
| $\mu$/mm$^{-1}$ | 0.088 |
| F(000) | 624.0 |
| Crystal size/mm$^3$ | 0.21 × 0.20 × 0.19 |
| Radiation | MoK$\alpha$ ($\lambda$ = 0.71073) |
| 2Θ range for data collection/° | 4.74 to 50 |
| Reflections collected | 7233 |
| Data/restraints/parameters | 1429/2/136 |
| Goodness-of-fit on F$^2$ | 1.051 |
| Final R indexes [I>=2σ (I)] | $R_1$ = 0.0529, w$R_2$ = 0.1679 |
| Final R indexes [all data] | $R_1$ = 0.0605, w$R_2$ = 0.1753 |
| Largest diff. peak/hole / e Å$^{-3}$ | 0.27/-0.23 |

**Table S2.** Lethality (%) of dechorionated embryos exposed to Tan-IIA at 12 hpf.

| | Solvent control | 1.0 μM | 5.0 μM | 10.0 μM | 20.0 μM | 50.0 μM |
|---|---|---|---|---|---|---|
| 12 hpf | 1.7 | 5.2 | 5.0 | 8.3 | 10.0 | 21.0 |

**Table S3.** Lethality (%) of chorionic embryos exposed to Tan-IIA at 12 hpf.

| | Solvent control | 1.5 μM | 3.0 μM | 6.0 μM | 12.0 μM | 24.0 μM |
|---|---|---|---|---|---|---|
| 24 hpf | 0.0 | 0.0 | 3.3 | 8.3 | 10.0 | 6.7 |

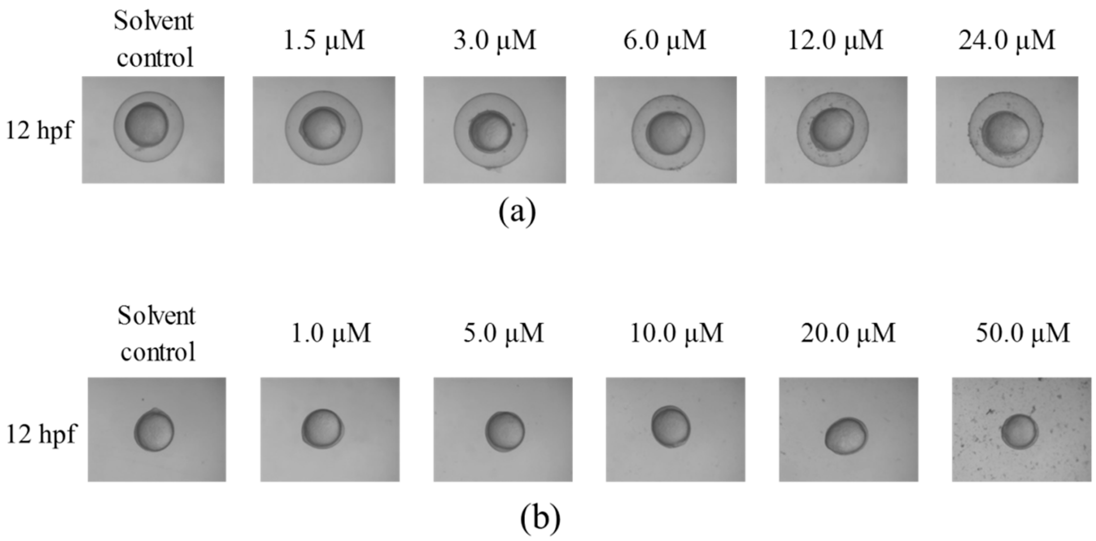


**Figure S1.** Morphology of zebrafish embryos exposed to Tan-IIA at 12 hpf. (a) Morphology of chorionic embryos; (b) Morphology of dechorionated embryos.

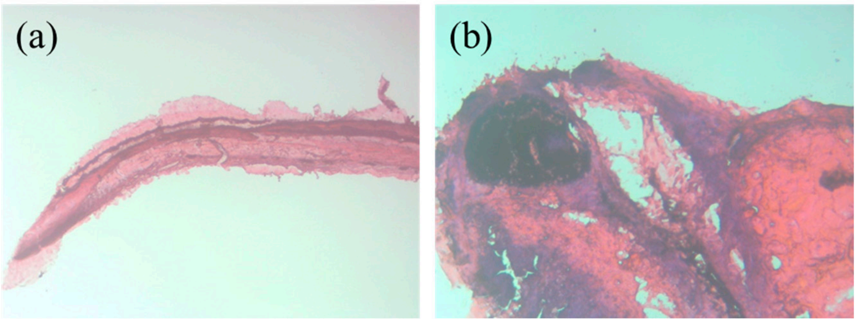


**Figure S2.** Longitudinal sections of scoliosis (a, ×10) and pericardial edema (b, ×40) with HE staining.

**Table S4.** Observations of teratogenic effects of dechorionated embryos at 48 hpf depending on concentration of Tan-IIA.

| | Solvent control | 1 μM | 5 μM | 10 μM | 20 μM | 50 μM |
|---|---|---|---|---|---|---|
| Scoliosis | | | | + | + | + |
| Tail autolysis | | | | + | + | + |
| Pericardial edema | | | | | + | + |
| Malformation of head | | | | | + | + |
| Malformation of eyes | | | | | + | + |
| Yolk sac edema | | | | | + | + |
| Growth retardation | | | | | | + |

**Table S5.** Observations of acute toxicity in dechorionated embryos exposed to Tan-IIA.

| | Exposure times | | | |
|---|---|---|---|---|
| | 24 hrs | 48 hrs | 72 hrs | 96 hrs |
| Coagulated embryos | + | + | + | |
| Lack of somite formation | + | + | | |
| Non-detachment of the tail | + | + | | |
| Lack of heartbeat | | + | + | + |
